# Functional characterization of the pheromone receptor OR67d within the genus *Drosophila*

**DOI:** 10.1016/j.isci.2026.118163

**Published:** 2026-11-09

**Authors:** Melissa Díaz-Morales, Mohammed A. Khallaf, Sukjin Steve Jang, Josefina del Mármol, Regina Stieber, Rongfeng Cui, Ibrahim Alali, Bill S. Hansson, Markus Knaden

**Affiliations:** 1 Department of Evolutionary Neuroethology, Max Planck Institute for Chemical Ecology, Jena, Germany; 2 Max Planck Center Next Generation Insect Chemical Ecology, Jena, Germany; 3 Lankester Botanical Garden, University of Costa Rica, Cartago, Costa Rica; 4 Department of Zoology and Entomology, Faculty of Science, Assiut University, Assiut, Egypt; 5 Department of Biological Chemistry and Molecular Pharmacology, Harvard Medical School, Boston, MA 02115, USA; 6 Howard Hughes Medical Institute, Boston, MA, USA; 7 School of Ecology & State Key laboratory of Biocontrol, Shenzhen Campus of Sun Yat-Sen University, Shenzhen, Guangzhou, China

**Keywords:** *Drosophila*, evolution, pheromone receptor, receptor tuning

## Abstract

Insects rely on chemosensory cues to mediate essential behaviors, including mate recognition. In *Drosophila melanogaster*, the odorant receptor OR67d detects the male-specific pheromone *cis*-vaccenyl acetate (cVA), initiating diverse behavioral responses. However, the evolutionary conservation and functional tuning of OR67d receptors across *Drosophila* species remain largely unexplored. Here, we characterize OR67d receptors from nine *Drosophila* species using phylogenetic analysis, transgenic expression, and electrophysiology. While most orthologs retained sensitivity to cVA, some exhibited broader tuning, responding to structurally similar acetates. Notably, *D. bipectinata* and *D. navojoa* responded to cVA despite not producing it, raising the possibility that cVA detection has been maintained independently of endogenous production, possibly as a heterospecific cue. Structural modeling revealed overall high sequence conservation yet identified variable regions within the putative binding pocket potentially underlying differences in ligand selectivity. These findings contribute to our understanding of pheromone receptor evolution and how chemical communication systems diversify across *Drosophila* species.

## Highlights

• OR67d function is largely conserved across diverse *Drosophila* species
• Some OR67d receptors broaden tuning to structurally related acetates
• Most OR67d orthologs retain cVA sensitivity, even in non-producing species
• Binding-pocket variation may underlie evolutionary shifts in ligand selectivity

## Graphical abstract

**Figure:**
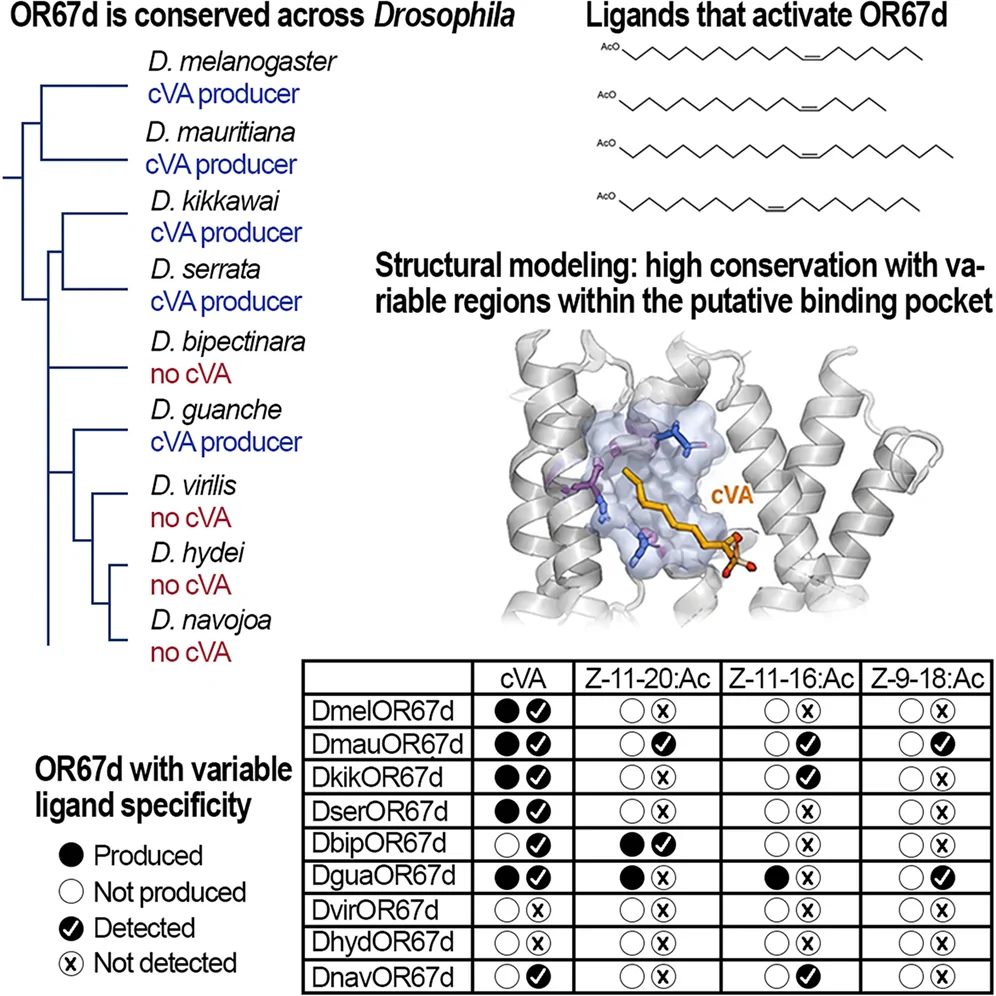

## Teaser

Biological sciences; Molecular biology; Evolutionary biology

## Introduction

Throughout their lives, insects encounter a vast array of chemical cues from their surroundings. These signals are critical for guiding behaviors such as choosing suitable egg-laying sites, identifying food sources, avoiding harmful substances, and selecting mating partners.^1^ To process this information, they use diverse chemosensory receptors that are essential for their survival, including odorant receptors (ORs). Unlike vertebrate ORs, insect ORs are not part of the G-protein-coupled receptor family; instead, the 7-transmembrane domains have an inverted topology with an intracellular N terminus and an extracellular C terminus.^2^ Moreover, insect ORs act as ligand-gated ion channels by forming a heteromeric complex with a highly conserved OR co-receptor, Orco.^2^^,^^3^ This ionotropic mode of action enables rapid and reliable odor-evoked responses that can be directly quantified.

In *Drosophila*, approximately 60 ortholog groups of OR genes have been identified,^4^ and many of these receptors have been functionally characterized using electrophysiological approaches.^5^^,^^6^ Notably, certain receptors have evolved to fulfill distinct ecological roles, leading to significant functional divergence.^7^ For instance, in *Drosophila*, the food odor-detecting receptor OR22a in *D. sechellia* exhibits a highly selective and sensitive response to methyl esters emitted by noni fruit, which serves as the primary host for this species.^8^ In contrast, homologous receptors OR22a/b in closely related *Drosophila* species show broader tuning, responding also to ethyl esters.^8^ By comparison, other food-related receptors like OR56a exhibit conserved functionality across *Drosophila* species, consistently recognizing geosmin, a microbial-associated compound that signals potential harm, indicating that evolutionary pressures have favored stability in some receptors due to the consistent ecological threat posed by harmful microbes.^9^ However, for these flies, the evolutionary modifications of pheromone receptors, which are key to intraspecies communication, remain underexplored.

Insect olfactory sensory neurons (OSNs) and their associated receptors, particularly those responding to sex or aggregation pheromones, are often highly specific.^7^^,^^10^ However, OSNs that detect non-pheromonal compounds can also exhibit high selectivity, especially for ubiquitous plant volatiles or other compounds critical for survival and reproduction.^1^^,^^7^^,^^9^^,^^11^^,^^12^ Such level of specificity in pheromone detection is thought to result from strong purifying selection acting on the corresponding receptors to maintain high fidelity in mate recognition systems. High selection pressures might help prevent mutations that could alter ligand specificity, which might then negatively impact reproductive success.^7^^,^^11^^,^^13^

Pheromone-responsive ORs provide an ideal candidate gene set for examining the evolutionary processes that drive sequence variation and functional adaptation for several reasons.^14^ First, pheromone ORs are generally expected to be fast-evolving chemosensory receptors, with new receptors emerging either by sequence variation or gene loss/duplication,^4^ to match the marked diversity of pheromones among closely related species and vice versa.^15^^,^^16^ Second, pheromone ORs are narrowly tuned to fly odors and govern robust and distinct behaviors via labeled-line circuitry.^17^^,^^18^ Last, only four pheromone ORs are localized in one particular class of sensilla; OR67d-expressing neurons in antennal trichoid 1 (at1); and OR47b-, OR65a/b/c-, and OR88a-expressing neurons in at4.^19^ This small number, compared with other non-pheromone-detecting ORs,^19^ makes a comprehensive evolutionary analysis feasible.

In *D. melanogaster*, the receptor OR67d is responsible for the detection of their sex pheromone (Z)-11-octadecen-1-yl acetate (*cis*-vaccenyl acetate, cVA).^15^^,^^17^^,^^20^^,^^21^^,^^22^ This pheromone is produced by at least 34 *Drosophila* species widely distributed across the phylogeny, making it the most prevalent male-specific compound in the genus.^15^ Additionally, many *Drosophila* species exhibited electrophysiological responses to cVA in OSNs housed in the at1 sensilla,^15^^,^^23^ suggesting that OR67d or its orthologs are functionally expressed in these neurons and contribute to cVA detection. In *D. bipectinata*, it was recently shown that the ortholog DbipOR67d not only detects cVA (despite this species not producing the pheromone) but also Z-11-eicosen-1-yl acetate, which is one of the compounds produced by *D. bipectinata* males.^24^

In *D. melanogaster*, cVA mediates multiple behaviors like reducing the sexual attractiveness of mated females,^17^^,^^25^^,^^26^ regulating male-male aggression,^22^^,^^27^ acting as an aphrodisiac for females,^17^^,^^28^ and promoting aggregation in the presence of food.^29^^,^^30^ However, little is known about the relevance of this pheromone in other *Drosophila* species and the functional properties of OR67d orthologs. Identifying the ligands of those orthologs will help to understand the functional evolution of the receptor and its corresponding ligands across *Drosophila* species and will shed light on its implications for sexual communication in the genus. In this study, we aimed to investigate whether OR67d orthologs across different clades in the *Drosophila* phylogeny are tuned to cVA or have changed their affinity to match the marked diversity of pheromones among *Drosophila* species.

## Results

### Phylogenetic analysis of OR67d receptors

To investigate whether the relationships among OR67d receptor orthologs and paralogs are conserved within the genus *Drosophila*, we built a phylogenetic tree based on 34 amino acid sequences of the receptor. The receptor for all species clustered together with the different species groups (Figure 1A). We then selected the sequences for the 9 species that we had available for electrophysiological experiments (see details in the STAR Methods) and calculated their amino acid identities. The identities within these 10 OR67d receptors range from 51% to 99% (Figure 1B). The highest identity similarity was observed between the two paralogs of *D. virilis* OR67d receptor (99.38%) and between those of the closely related species *D. melanogaster* and *D. mauritiana* (99.23%) (Figure 1B). Notably, branch-specific adaptive branch-site random effects likelihood (aBSREL) analyses detected significant episodic positive selection only on the *D. mauritiana* lineage (likelihood-ratio test = 11.52, Holm-Bonferroni-corrected *p* = 0.018), with all other lineages showing no significant evidence of positive selection after multiple-testing correction (Table S1).

**Figure 1:**
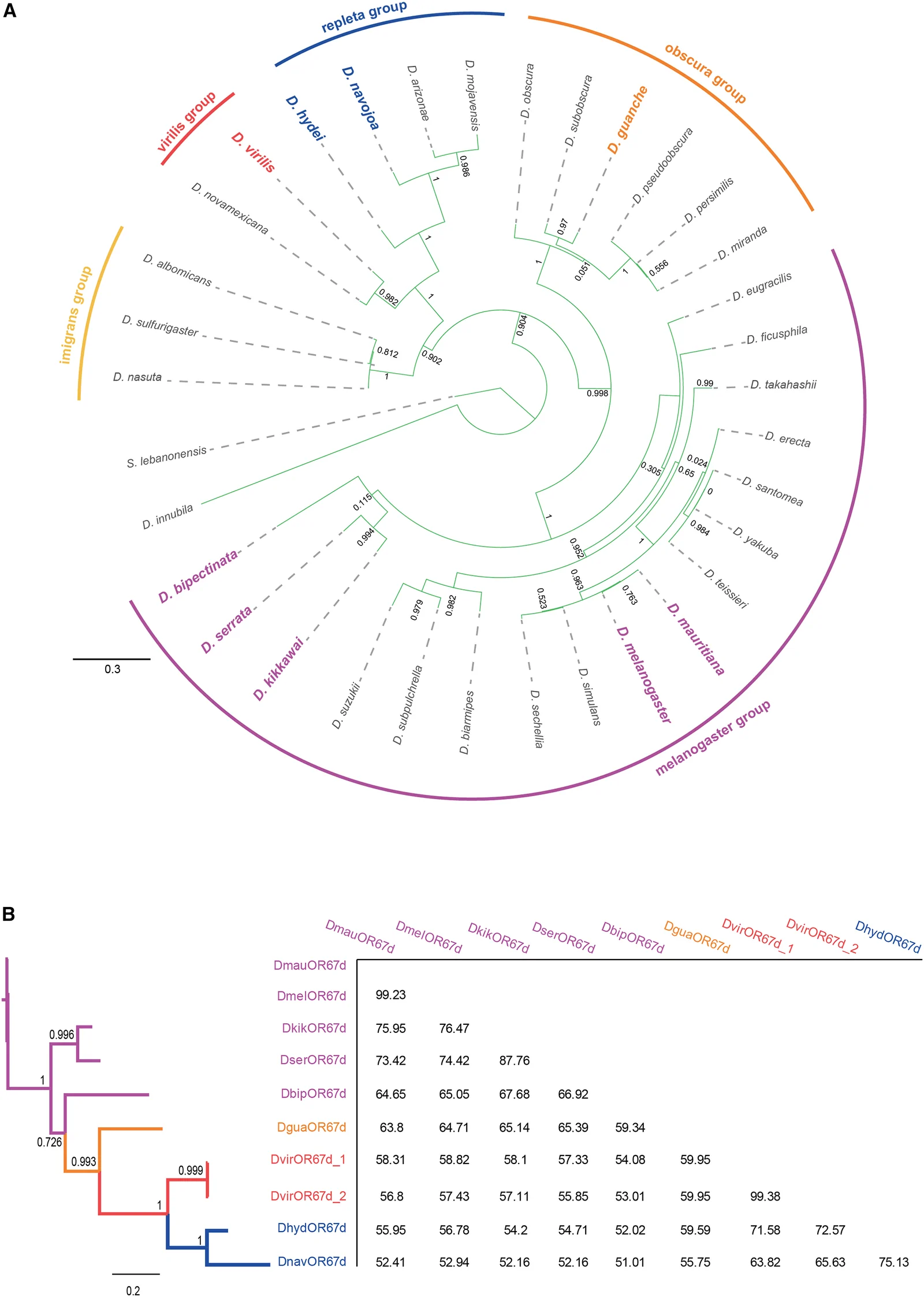
Phylogenetic relationships within OR67d receptors of *Drosophila* species (A) Maximum-likelihood phylogeny based on the amino acid sequences of the OR67d receptor for 34 drosophilid flies. The ortholog of *Scaptodrosophila lebanonensis* (Drosophilidae: *Colocasiomyini*) was used as an outgroup. Support values were calculated using the Shimodaira-Hasegawa test implemented in FastTree. The clustering of species matches the phylogenetic groups of the genus.^15^ Species highlighted in color indicate those used later for electrophysiological experiments. (B) Distance matrix showing the % identity (percentage of bases/residues that are identical) based on multiple alignment using fast Fourier transform (MAFFT) alignment of the 10 selected receptors. Scale bars represent amino acids substitutions per site (0.3 in A and 0.2 in B).

### Responses of OR67d receptors to male-specific compounds

To investigate the functional responses of OR67d receptors, we generated transgenic *D. melanogaster* flies expressing each receptor individually *in vivo* within the at1 “decoder” neuron, which lacks its native OR67d receptor.^31^ These include the orthologs DbipOR67d, DguaOR67d, DhydOR67d, DkikOR67d, DmauOR67d, DmelOR67d, DnavOR67d, and DserOR67d and the paralogs DvirOR67d1 and DvirOR67d2. We then conducted single-sensillum recordings (SSRs) on these transgenic flies using a panel of 36 male-specific *Drosophila* compounds (Figure 2A) identified in a recent study.^15^ We found responses to only 4 male-specific compounds that are structurally very similar to each other, cVA (Z11-18:Ac), (Z)-11-hexadecen-1-yl acetate (Z11-16:Ac), (Z)-11-eicosen-1-yl acetate (Z11-20:Ac), and (Z)-9-octadecen-1-yl acetate (Z9-18:Ac) (Figure 2B). Of the ten screened receptors, seven showed significantly positive responses to cVA (Figures 2C–2I), including *D. bipectinata* and *D. navojoa* that do not produce it. The *D. mauritiana* receptor was the only one that responded to four acetates, although even here the strongest response was to cVA, i.e., the only one of those compounds that is also produced by this species (Figure 2C). In contrast, the receptors of *D. melanogaster* and *D. serrata* responded exclusively to cVA (Figures 2D and 2E). The OR67d ortholog of *D. kikkawai* showed similarly positive responses to cVA (produced by this species) and Z11-16:Ac (not produced; Figure 2F), while the *D. bipectinata* receptor responded to cVA (not produced) and Z11-20:Ac (produced; Figure 2G). In *D. guanche*, we observed responses to cVA (produced) and Z9-18:Ac (not produced; Figure 2H), and the *D. navojoa* receptor exhibited positive responses to cVA and Z11-16:Ac, neither of which is produced by this species (Figure 2I). However, the receptors of *D. hydei* and *D. virilis* did not respond to any of the tested compounds under these conditions (Figures 2J–2L).

**Figure 2:**
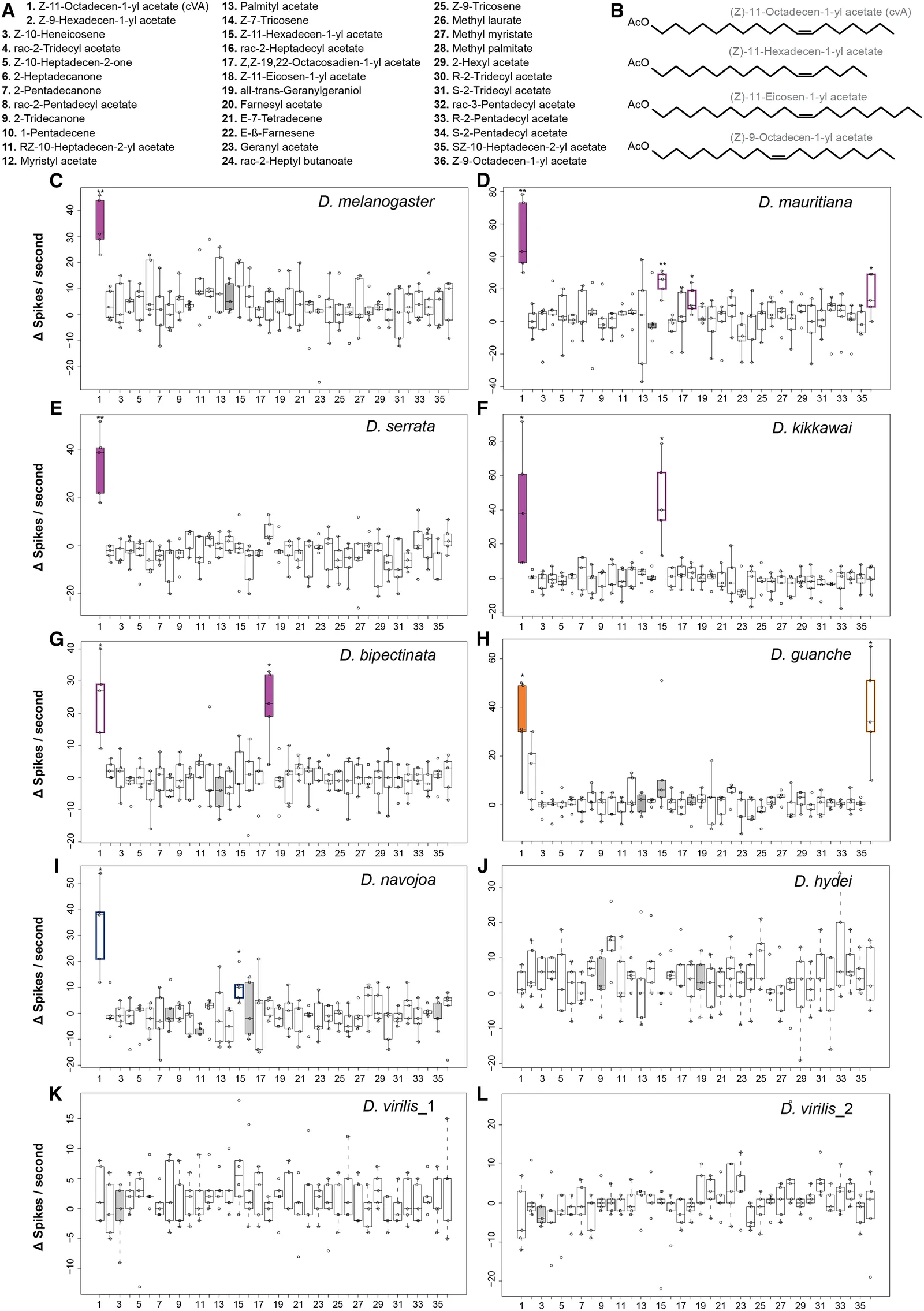
SSR responses from *D. melanogaster* at1 OSNs and *D. melanogaster* at1 OSNs that lack their own receptor but ectopically express the receptors of *D. mauritiana*, *D. kikkawai*, *D. serrata*, *D. bipectinata*, *D. guanche*, *D. navojoa*, *D. hydei*, and *D. virilis* in the at1 neuron (A) List of the tested *Drosophila* male-specific compounds. (B) Chemical structure of the four acetates for which the receptors showed significantly positive responses (the chemical structure of the other 32 tested compounds is provided in Figure S1). (C) OSNs in *D. melanogaster* Canton-S (CS) at1 sensilla respond to cVA. (D) The receptor DmauOR67d responds to Z11-18:Ac (cVA), Z11-16:Ac, Z11-20:Ac, and Z9-18:Ac. (E) The receptor DserOR67d responds to cVA. (F) The receptor DkikOR67d responds to cVA and Z11-16:Ac. (G) The receptor DbipOR67d responds to cVA and Z11-20:Ac. (H) The receptor DguaOR67d responds to cVA and Z9-18:Ac. (I) The receptor DnavOR67d responds to cVA and Z11-16:Ac. (J–L) The receptors DhydOR67d, DvirOR67d1, and DvirOR67d2, respectively, did not show significantly positive responses to the tested compounds. For all tested flies, the solvent dichloromethane (DCM) was used as a control. The *y* axes represent Δ spikes/second; the *x* axes represent the tested compounds. Color-filled boxplots represent statistically significant responses to compounds produced by each of these species. Color-lined boxplots represent statistically significant responses to compounds not produced by these species. Gray-filled boxplots represent non-statistically significant responses to compounds produced by these species. Purple represents species of the “*melanogaster* group,” orange represents those of the “*obscura* group,” and blue represents those of the “*repleta* group.” Asterisks indicate the statistical significances of comparisons between each compound and the solvent control (⁎*p* < 0.05; ⁎⁎*p* < 0.01; *n* = 5–6; one sample *t* test).

### Sequence divergence in the putative OR67d binding pocket

To examine potential sequence patterns that capture the divergence in pheromone sensitivity across the clade, we mapped sequence conservation onto an AlphaFold 3-predicted structure of DmelOR67d bound to cVA.^32^ The predicted structure placed the polar acetate functional group in a cavity lined by S1, S2, and S4, and the hydrophobic tail end near S3 and S6 (Figure 3A). At the acetate-facing end of the pocket, two polar residues—Asn29 and Thr93—are within distances that allow them to form hydrogen bonds (Figure 3B). Looking at the conservation of these residues (Figure 3C), the receptors DhydOR67d, DnavOR67d, DvirOR67d1, and DvirOR67d2 diverge from the rest, with Asn29 being a Thr (DvirOR67d2 has a truncation instead) and Thr93 being a Cys. The receptors of *D. hydei* and the two paralogs of *D. virilis* have no response to cVA, suggesting that these residues may play an important role in recognition of the acetate group. Meanwhile, the OR67d receptor from *D. navojoa* still displays robust sensitivity to cVA, indicating that these interactions can be bypassed during recognition of cVA. On the other end of the pocket, the hydrophobic tail sits at a junction between the S3 and S6 helices (Figure 3D). Previous structural studies of heteromeric insect ORs have shown that interactions between the ligand and residues lining the binding pocket from S3 to S6 helices play an essential role in receptor activation.^33^^,^^34^^,^^35^^,^^36^ Across the *Drosophila* OR67d receptors, residues in the S3 helix were notably variable (Figure 3E), hinting that mutations in this region may tune the sensitivity of different OR67d variants to different long-aliphatic-chain acetate pheromone ligands. While the precise binding pose of cVA cannot be reliably inferred given the predicted structure, these observations provide valuable insights to narrow down the potential regions of the binding pocket that have diverged across species to retune their pheromone selectivity.

**Figure 3:**
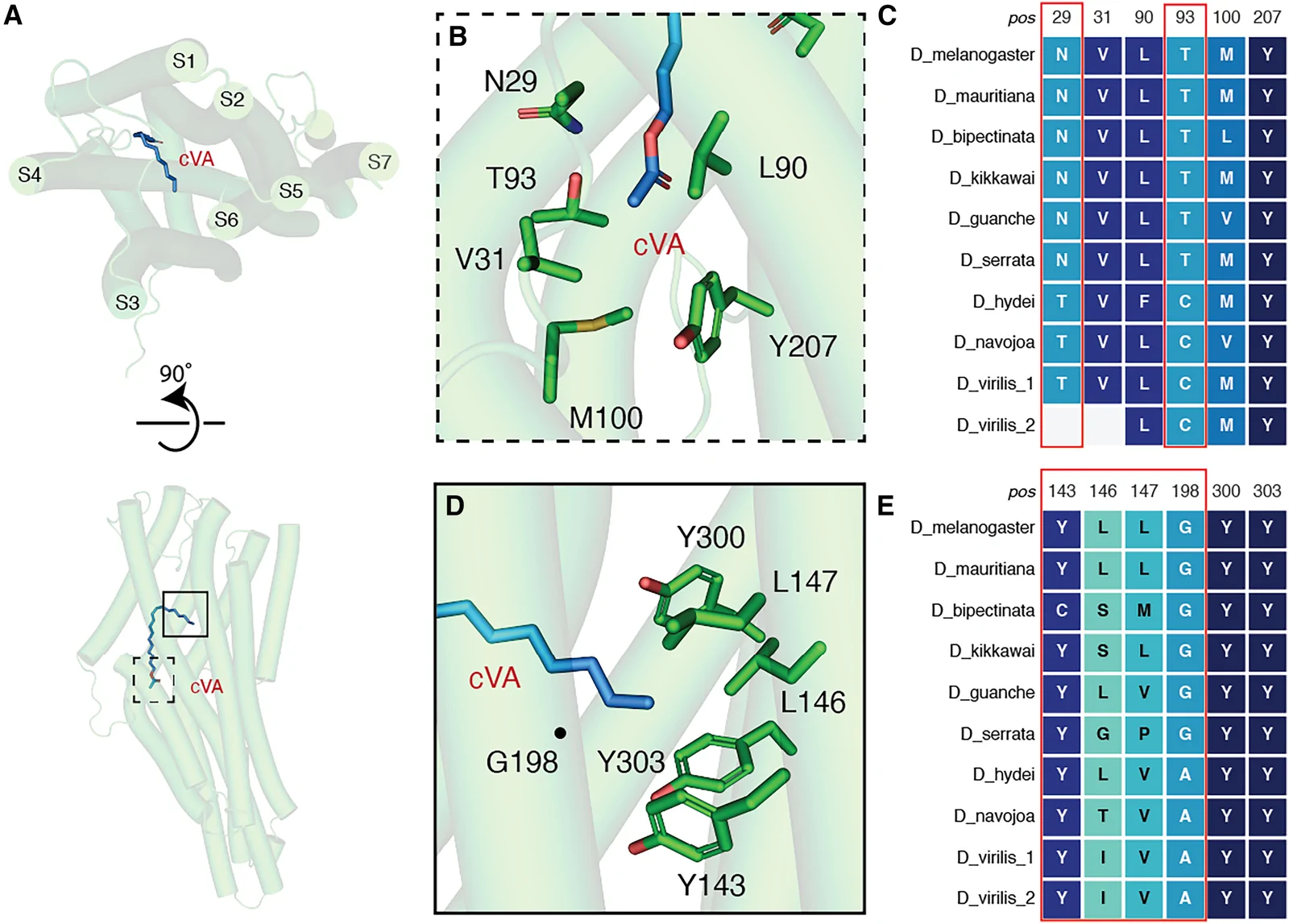
Putative binding of cVA to OR67d receptors of *Drosophila* (A) Side and extracellular views of the predicted structure of *D. melanogaster* OR67d, showing a putative binding pose of the pheromone cVA. (B and C) Close-up view of binding-pocket residues surrounding the acetate group (B) and sequence alignments of the corresponding residues across *Drosophila* OR67d homologs (C). (D and E) Close-up view of binding-pocket residues surrounding the hydrophobic tail (D) and sequence alignments of the corresponding residues across *Drosophila* OR67d homologs (E). In the sequence alignment, dark blue represents higher conservation, and light blue represents lower conservation. The red box highlights sites of highest variability.

## Discussion

The pheromone receptor OR67d, which detects cVA in *D. melanogaster*, has been extensively studied due to the ecological significance of this pheromone in the species.^17^^,^^22^^,^^25^^,^^26^^,^^27^^,^^28^^,^^30^ In *D. bipectinata*, OR67d similarly detects both cVA and Z11-20:Ac, demonstrating that OR67d orthologs can differ in ligand sensitivity while remaining functionally active.^24^ In this study, we functionally characterized eight additional OR67d receptors from *Drosophila* species that are widely distributed across the phylogeny of the genus, finding that most of them conserve their tuning to cVA, with some of them gaining functionality by detecting additional structurally similar acetates.

Most of the tested OR67d receptors retain sensitivity to cVA, and the corresponding species produce this pheromone, regardless of its specific ecological function in each species.^15^ However, *D. bipectinata* and *D. navojoa* are unique in retaining the ability to detect cVA without producing it themselves.^15^ This suggests that cVA detection may have been evolutionarily conserved despite the loss of endogenous pheromone production, potentially because the compound still conveys ecologically relevant information. Notably, in *D. navojoa*, cVA acts as an antiaphrodisiac by reducing male attractiveness, an effect opposite to its effect in *D. melanogaster*.^15^ A similar effect of cVA has been reported in other members of the “*repleta* group,” including the cactophilic species of the *D. mojavensis* complex.^15^^,^^37^ These observations raise the possibility that, in some species, cVA no longer functions primarily as a conspecific sexual signal but rather as an environmental or interspecific cue, which could be associated with competitor presence, mating activity, or occupied breeding substrates. The conservation of cVA detection may reflect the ecological value of monitoring heterospecific signals, while the behavioral valence associated with this pheromone has diverged across species.

As expected, expression of the Or67d gene in OSNs housed in at1 sensilla is highly conserved across the *melanogaster* species group, as evidenced by the positive responses of OR67d receptors to cVA and similar acetates in these species, consistent with previous SSR recordings from at1 sensilla in wild-type flies.^15^ A similar pattern was also observed in the distantly related *repleta* species group, specifically in *D. navojoa*. However, the lack of responses from the OR67d ortholog in *D. hydei* (another member of the *repleta* group) and from the OR67d paralogs in *D. virilis* suggests that the conservation of the Or67d gene expression is not persistent across all distantly related species. Although this pattern is consistent with altered ligand tuning, further analyses of receptor expression and localization are needed to exclude reduced functional expression as a contributing factor. One possible explanation is that their ligands were not included in the odor panel. Alternatively, receptor activation could depend on molecular components absent or altered in the heterologous expression system, such as species-specific sensory neuron membrane proteins (SNMPs) and odorant binding proteins (OBPs), which are known to facilitate odorant-receptor interactions.^29^^,^^38^^,^^39^ However, the at1-based heterologous expression system used here has been shown to support functional expression of a wide range of insect receptors, including pheromone receptors from distantly related taxa.^40^^,^^41^^,^^42^^,^^43^ Moreover, the lack of cVA responses in at1 sensilla of wild-type flies of *D. hydei* and *D. virilis* has been previously documented. Therefore, the lack of responses observed for these OR67d receptors likely reflects genuine differences in ligand tuning rather than technical limitations.

At the molecular level, changes in ligand specificity can arise from non-synonymous substitutions, insertions, or deletions that alter the geometry or physicochemical properties of the ligand-binding pocket.^30^^,^^44^^,^^45^ Despite the overall high sequence conservation among OR67d receptors, we identified regions of high variability that map to putative ligand-interacting domains. A set of residues in the S3 helix and two polar residues, Asn29 and Thr93, in *D. melanogaster* exhibit variability across the species. Recently, cryo-electron microscopy (cryo-EM) structures of the OR67d receptors from *D. melanogaster* and *D. bipectinata* were solved, providing exciting color to the functional data presented in this work.^46^ The experimental structures display strikingly similar binding poses of cVA compared to the AlphaFold 3-predicted structure. The structures capture the acetate group of cVA positioned deep into the pocket formed by S1, S2, and S4 and the hydrophobic tail facing the S3–S6 helices. The acetate group forms two key hydrogen bonds with the two polar residues (Asn29 and Thr93 in *D. melanogaster*), and mutation of each of these residues to Ala abolishes their activity. Furthermore, the work suggests two key mutations in *D. bipectinata* (Tyr143 to Cys and Leu147 to Met in the S3 helix) compared to *D*. *melanogaster* that enable the *D. bipectinata* OR67d receptor to selectively detect Z-11-eicosen-1-yl acetate. These results point to sequence divergence in these two specific regions of the ligand-binding pocket as retuning pheromone selectivity across these *Drosophila* species. Finally, the bimodal binding mode of OR67d homologs and their cVA derivatives draws clear parallel with the binding mode of bombykal in *Bombyx mori* odorant receptor 3 (BmOR3), hinting a general strategy of odorant recognition for long-chain aliphatic pheromones.^34^

### Limitations of the study

Our results show that OR67d receptors range from highly selective cVA responses to broader tuning or no detectable responses to the tested odor panel, providing a comparative framework for OR67d function across *Drosophila*. However, broader ligand panels, additional species, and quantitative dose-response analyses will be needed to better resolve receptor tuning and its evolutionary significance.

The structural analyses should also be interpreted cautiously. AlphaFold predictions were used as hypothesis-generating tools rather than definitive evidence of ligand-binding mechanisms. The predicted binding pocket and variable residues identify candidate regions for future validation through structural studies, molecular dynamics, and targeted mutagenesis. Behavioral and genetic approaches, including knockout experiments, will further be needed to establish the *in vivo* relevance of receptor differences. Finally, although the lack of responses in *D. hydei* and *D. virilis* likely reflects differences in ligand tuning, technical limitations cannot be completely excluded.

## Resource availability

### Lead contact

Requests for further information and resources should be directed to and will be fulfilled by the lead contact, Markus Knaden (mknaden@ice.mpg.de).

### Materials availability

Fly lines generated in this study have been deposited in the fly stock at the Max Planck Institute for Chemical Ecology under the catalog numbers 2024, 2025, 2027, 2028, 2029, 2030, 2074, 2077, and 2078.

### Data and code availability

• OR67d nucleotide and protein sequences, sequence alignments, the phylogenetic tree shown in Figure 1, dN/dS analysis results, and raw single-sensillum recording data have been deposited in Edmond (Max Planck Digital Library): https://doi.org/10.17617/3.GHFNWQ. These data are publicly available. The persistent identifier is listed in the key resources table.
• This paper does not report original code.
• Any additional information required to reanalyze the data reported in this paper is available from the lead contact upon request.

## Acknowledgments

We thank Lea Uhde for helping with fly rearing and electrophysiology. We are grateful to Silke Trautheim, Roland Spieβ, Manal Alali, and Angela Lehmann for their technical support. This research was funded by the Max Planck Society and the 10.13039/501100005298University of Costa Rica through a doctoral fellowship. The study was funded by the Max Planck Society (B.S.H.) and the Max Planck Centre nGICE (Next Generation Insect Chemical Ecology; to B.S.H. and M.K.). S.S.J. was supported by the Howard Hughes Medical Institute. J.d.M. is a Freeman Hrabowski Scholar of the Howard Hughes Medical Institute.

## Author contributions

Conceptualization, M.D.-M., M.A.K., B.S.H., and M.K.; data curation, M.D.-M., M.A.K., S.S.J., and R.S.; formal analysis, M.D.-M., M.A.K., and S.S.J.; methodology, M.D.-M., M.A.K., R.S., and I.A.; resources, S.S.J., J.d.M., and R.C.; supervision, J.d.M., B.S.H., and M.K.; visualization, M.D.-M., M.A.K., S.S.J., and J.d.M.; writing – original draft, M.D.-M. and M.A.K.; writing – review and editing, all authors.

## Declaration of interests

The authors have no conflict of interests to declare.

## Declaration of generative AI and AI-assisted technologies in the manuscript preparation process

During the preparation of this work, the authors used ChatGPT, DeepL, and Illustrator to assist with graphical illustrations and grammar, and spelling checks throughout the work. After using these tools, the authors reviewed and edited the content as needed and take full responsibility for the content of the published article.

## STAR★Methods

### Key resources table

**Table:** 

REAGENT or RESOURCE	SOURCE	IDENTIFIER
**Chemicals, peptides, and recombinant proteins**		

(Z)-11-Octadecen-1-yl acetate	AK Scientific, Inc.	CAS: 6186–98–7
(Z)-9-Hexadecen-1-yl acetate	Khallaf et al.^15^	N/A
(Z)-10-Heneicosene	Khallaf et al.^15^	N/A
rac-2-Tridecyl acetate	Khallaf et al.^15^	N/A
(Z)-10-Heptadecen-2-one	Khallaf et al.^15^	N/A
2-Heptadecanone	Khallaf et al.^15^	N/A
2-Pentadecanone	Sigma-Aldrich	CAS: 2345–28–0
rac-2-Pentadecyl acetate	Khallaf et al.^15^	N/A
2-Tridecanone	Sigma-Aldrich	CAS: 593–08–8
1-Pentadecene	Sigma-Aldrich	CAS: 13360–61–7
R-(Z)-10-Heptadecen-2-yl acetate	Khallaf et al.^15^	N/A
Myristyl acetate	AKos Consulting and Solutions	CAS: 638–59–5
Palmityl acetate	AKos Consulting and Solutions	CAS: 629–70–9
(Z)-7-Tricosene	AKos Consulting and Solutions	CAS: 52078–57–6
(Z)-11-Hexadecen-1-yl acetate	Khallaf et al.^15^	N/A
rac-2-Heptadecyl acetate	Khallaf et al.^15^	N/A
(Z,Z)-19,22-Octacosadien-1-yl acetate	Khallaf et al.^15^	N/A
(Z)-11-Eicosen-1-yl acetate	Khallaf et al.^15^	N/A
All-trans-Geranylgeraniol	Sigma-Aldrich	CAS: 24034–73–9
Farnesyl acetate	Khallaf et al.^15^	N/A
(E)-7-Tetradecene	Alfa Chemistry	CAS: 41446–63–3
(E)-β-Farnesene	Sigma-Aldrich	CAS: 18794–84–8
Geranyl acetate	Sigma-Aldrich	CAS: 105–87–3
rac-2-Heptyl butanoate	Alfa Chemistry	CAS: 39026–94–3
(Z)-9-Tricosene	Sigma-Aldrich	CAS: 27519–02–4
Methyl laurate	Sigma-Aldrich	CAS: 111–82–0
Methyl myristate	Sigma-Aldrich	CAS: 124–10–7
Methyl palmitate	Sigma-Aldrich	CAS: 112–39–0
2-Hexyl acetate	Khallaf et al.^15^	N/A
R-2-Tridecyl acetate	Khallaf et al.^15^	N/A
S-2-Tridecyl acetate	Khallaf et al.^15^	N/A
rac-3-Pentadecyl acetate	Khallaf et al.^15^	N/A
R-2-Pentadecyl acetate	Khallaf et al.^15^	N/A
S-2-Pentadecyl acetate	Khallaf et al.^15^	N/A
S-(Z)-10-Heptadecen-2-yl acetate	Khallaf et al.^15^	N/A
(Z)-9-Octadecen-1-yl acetate	Khallaf et al.^15^	N/A
(Z)-7-Pentacosene	Biomol GmbH	CAS: 63623–49–4

**Deposited data**		

dN/dS analyses (nucleotide and protein alignments, and results)	This study	https://doi.org/10.17617/3.GHFNWQ
*D. bipectinata* OR67d sequence	This study	https://doi.org/10.17617/3.GHFNWQ
*D. serrata* OR67d sequence	This study	https://doi.org/10.17617/3.GHFNWQ
*D. kikkawai* OR67d sequence	This study	https://doi.org/10.17617/3.GHFNWQ
*D. mauritiana* OR67d sequence	This study	https://doi.org/10.17617/3.GHFNWQ
*D. guanche* OR67d sequence	This study	https://doi.org/10.17617/3.GHFNWQ
*D. navojoa* OR67d sequence	This study	https://doi.org/10.17617/3.GHFNWQ
*D. hydei* OR67d sequence	This study	https://doi.org/10.17617/3.GHFNWQ
*D. virilis* OR67d sequence	This study	https://doi.org/10.17617/3.GHFNWQ
*D. melanogaster* OR67d sequence	NCBI GenBank	RefSeq: NP_648390.2
Phylogenetic tree in Figure 1	This study	https://doi.org/10.17617/3.GHFNWQ
Raw single-sensillum recording (SSR) data	This study	https://doi.org/10.17617/3.GHFNWQ

**Experimental models: Organisms/strains**		

*Drosophila melanogaster*	This study	N/A
*D. melanogaster* expressing DbipOR67d (+; UAS-DbipOr67d/UAS-DbipOr67d; Or67d-Gal4/Or67d-Gal4)	This study	N/A
*D. melanogaster* expressing DguaOR67d (+; UAS-DguaOr67d/UAS-DguaOr67d; Or67d-Gal4/Or67d-Gal4)	This study	N/A
*D. melanogaster* expressing DhydOR67d (+; UAS-DhydOr67d/UAS-DhydOr67d; Or67d-Gal4/Or67d-Gal4)	This study	N/A
*D. melanogaster* expressing DkikOR67d (+; UAS-DkikOr67d/UAS-DkikOr67d; Or67d-Gal4/Or67d-Gal4)	This study	N/A
*D. melanogaster* expressing DmauOR67d (+; UAS-DmauOr67d/UAS-DmauOr67d; Or67d-Gal4/Or67d-Gal4)	This study	N/A
*D. melanogaster* expressing DnavOR67d (+; UAS-DnavOr67d/UAS-DnavOr67d; Or67d-Gal4/Or67d-Gal4)	This study	N/A
*D. melanogaster* expressing DserOR67d (+; UAS-DserOr67d/UAS-DserOr67d; Or67d-Gal4/Or67d-Gal4)	This study	N/A
*D. melanogaster* expressing Dvir1OR67d (+; UAS-Dvir1Or67d/UAS-Dvir1Or67d; Or67d-Gal4/Or67d-Gal4)	This study	N/A
*D. melanogaster* expressing Dvir2OR67d (+; UAS-Dvir2Or67d/UAS-Dvir2Or67d; Or67d-Gal4/Or67d-Gal4)	This study	N/A

**Software and algorithms**		

R version 4.3.0	RRID: SCR_001905, R Core Team^47^	https://www.R-project.org/
Geneious Prime 2004.0.5	Dotmatics	https://www.geneious.com/
AutoSpike version 3.10	Syntech	https://www.ockenfels-syntech.com/
AlphaFold 3	Abramson et al.^32^	https://alphafoldserver.com/
Maestro	Schrödinger	https://www.schrodinger.com/platform/products/maestro/

### Experimental model and study participant details

#### Flies

We generated the transgenic fly lines expressing the receptors DbipOR67d, DguaOR67d, DhydOR67d, DkikOR67d, DmauOR67d, DnavOR67d, DserOR67d, DvirOR67d1, and DvirOR67d2 ectopically in *D. melanogaster* wild-type flies. All flies used for electrophysiological experiments were bred on conventional cornmeal agar medium under a 12h light and 12h dark cycle at 25°C and 70% humidity. Care and treatment of all flies complied with all relevant ethical regulations.

### Method details

#### Phylogenetics of OR67d receptors

The phylogenetic relationships of the OR67d receptor were analyzed based on the amino acid sequences from 34 species of Drosophilidae (Table S2). The sequence alignment was performed using MAFFT v7.490, and a maximum-likelihood tree rooted with the ortholog receptor sequence of *Scaptodrosophila lebanonensis* (Drosophilidae: Colocasiomyini) was built using FastTree 2.1.11 implemented in Geneious Prime 2004.0.5. Support values were calculated using the Shimodaira–Hasegawa test implemented in FastTree. A posterior MAFFT alignment of the 10 OR67d receptors used for electrophysiological experiments was used to calculate the distance matrix showing the % Identity (percentage of bases/residues which are identical) among them.

#### Detection of branch-specific positive selection

Coding DNA sequences were aligned using a protein-guided codon alignment approach to preserve the reading frames and codon structure during sequence comparison. Branch-specific episodic positive selection was tested using the aBSREL model implemented in HyPhy v2.5 and accessed through the DataMonkey web servers.^48^ The aBSREL model tests each branch of a phylogenetic tree independently for evidence of episodic diversifying selection, allowing the strength of selection to vary both among branches and codon sites within a branch. The model estimates the ratio of nonsynonymous to synonymous substitution rates (ω = dN/dS), where ω > 1 indicates positive selection. Evidence for positive selection on individual branches was evaluated using likelihood ratio tests comparing models with and without positively selected site classes. Analyses were conducted using a maximum-likelihood phylogeny and the universal genetic code. To account for multiple testing across branches, statistical significance was determined using the Holm–Bonferroni correction.

#### Cloning of OR67d receptors and transgenic expression in *D. melanogaster* at1 neuron

The species selected for the cloning of the OR67d receptor and its transgenic expression were chosen to cover several major phylogenetic groups within *Drosophila*. Two *D. virilis* paralogs were included to examine possible functional divergence after gene duplication. However, practical requirements, including access to biological material and successful cloning of a complete *Or67d* sequence, also affected the final selection (i.e., the ‘imigrans group’ is not represented in the data). To clone the full-length coding sequence of the seven OR67d orthologs (DbipOR67d, DguaOR67d, DhydOR67d, DkikOR67d, DmauOR67d, DnavOR67d, DserOR67d) and two paralogs (DvirOR67d1, and DvirOR67d2), total antennal RNA from approximately 100 flies for each species was prepared, and cDNA was synthesized using the Superscript III Reverse Transcriptase kit (Thermo Fisher Scientific, Germany). Each *Or67d* gene was amplified by using PCR with the following gene-specific primers: DxOr67d-Forward: 5′ ATGTCCAAATTAAATAAAAAATTAACACCCGTTGAC 3′; DxOr67d-Reverse: 5′ CTATTTTTCCATTCCCAAGTAGGTCAGC 3′. The resulting construct of the PCR reaction was purified and T:A cloned into pCR^TM^8/GW/TOPO (Thermo Fisher Scientific, Germany). Sanger sequencing confirmed the sequence (Eurofins Genomics Germany GmbH and the Department of Insect Symbiosis, Max Planck Institute for Chemical Ecology, Jena).

The construct was transferred to the destination vector pUASg.attB (provided by J. Bischof, FLYORF Injection, Zürich, Switzerland) following Gonzalez et al.^31^ Subsequently, FlyORF Injection performed the germline transformation of *D. melanogaster* with the prepared plasmid, using the PhiC31 integration system. The vector was inserted into chromosome II, fly strain attP40: 2L, 25C6 to produce the genotype +; UAS- DxOr67/SM6a; +.

To generate the experimental fly line (+; UAS-*Dx*Or67d/UAS-*Dx*Or67d; Or67d-Gal4/Or67d-Gal4) expressing *DxOr67d* under the regulation of *Or67d* in *D. melanogaster*, the fly lines +; UAS-*Dx*Or67/SM6a,yw; BL/Cyo; Tm2/Tm6b (obtained from Bloomington Stock Center, no. BL3704) and +; BL/Cyo; Or67d-Gal4 no.1/Tm6b (obtained from IMP, Vienna, no. M1139) were used to perform genetic crosses following Gonzalez et al.^31^ This resulted in flies that do not express the endogenous *D. melanogaster Or67d* gene anymore, but instead express the receptor gene of interest.

#### Electrophysiology

SSR was performed on at1 sensilla following the protocol described by Olsson and Hansson^49^ with modifications described in Diaz-Morales et al.^24^ Briefly, adult male and female flies (6–10 days old) were immobilized in a 100 μL plastic pipette tip. The third antennal segment was placed in a stable position on a glass coverslip in a ventrolateral position, and the funiculus of the antenna was fixed with a sharpened glass capillary.

Stimulus cartridges were prepared with 10 μL of the diluted odor pipetted onto a 1 cm ⌀ piece of filter paper (Rotilabo type 601A, Carl Roth, Germany), placed inside a Pasteur pipette using as lid a 1 mL plastic pipette clogged with wax to prevent evaporation. We used dilutions in DCM (Sigma-Aldrich, Germany) at a concentration of 10^−3^ (v/v).^15^^,^^24^

The antenna of the fly was stimulated by a 0.5 s air pulse (0.5 lpm) through the stimulation cartridge located ca. 10 mm from the antenna.^50^ The entire odorant panel (see key resources table) was tested on one to three sensilla per fly. The extracellular signals originating from the OSNs were measured with a tungsten wire electrode inserted in the base of a sensillum and a reference electrode into the eye. Signals were amplified (Syntech Universal AC/DC Probe; Syntech), sampled (9,600.0 samples/s), and filtered (300–3000 Hz with 50/60 Hz suppression) via USB-IDAC connection to a computer (Syntech).

Action potentials were extracted using AutoSpike software, version 3.10 (Syntech). Neuron activities were recorded 2 s before stimulation and 3 s after stimulation. Neural responses were calculated as the increase (or decrease) in the action potential frequency (spikes/s) relative to the pre-stimulus frequency by manually counting the number of spikes 1 s before and 1 s after the stimulus.

#### Prediction of OR67d structure

The predicted structure of DmelOR67d bound to cVA was generated using AlphaFold3.^32^ OR67d orthologs from nine *Drosophila* species (*D. melanogaster, D. mauritiana, D. bipectinata, D. kikkawai, D. guanche, D. serrata, D. hydei, D. navojoa,* and *D. virilis*; ten sequences including two *D. virilis* variants) were aligned at the amino acid level with MAFFT v7.526. Per-column conservation was calculated as the fraction of sequences matching the most common (consensus) residue at each aligned position, and residue positions are numbered relative to the *D. melanogaster* OR67d sequence.

### Quantification and statistical analysis

For the electrophysiological experiments, the normality test was first assessed on datasets using a Shapiro test. Statistical analyses (see the corresponding legends of each figure for details) and preliminary figures were conducted using R version 4.3.0.^47^ Figures were then processed with Adobe Illustrator CS5.

## Supplemental information

Document S1. Figure S1

Table S1. Branch-specific episodic positive selection analysis using aBSREL

Table S2. Sequences used for phylogenetic analyses
