# Supplementary material for: Functional characterization of the pheromone receptor OR67d within the genus Drosophila
Source: iScience. 2026 Nov 9;29(11):118163. doi: 10.1016/j.isci.2026.118163 (PMC13760699; doi:10.1016/j.isci.2026.118163)
Supplement: Supplementary file 1 [file mmc1.pdf]

iScience, Volume *29*

Supplemental information

Functional characterization of the pheromone receptor OR67d within the genus *Drosophila*

Melissa Díaz-Morales, Mohammed A. Khallaf, Sukjin Steve Jang, Josefina del Mármol, Regina Stieber, Rongfeng Cui, Ibrahim Alali, Bill S. Hansson, and Markus Knaden

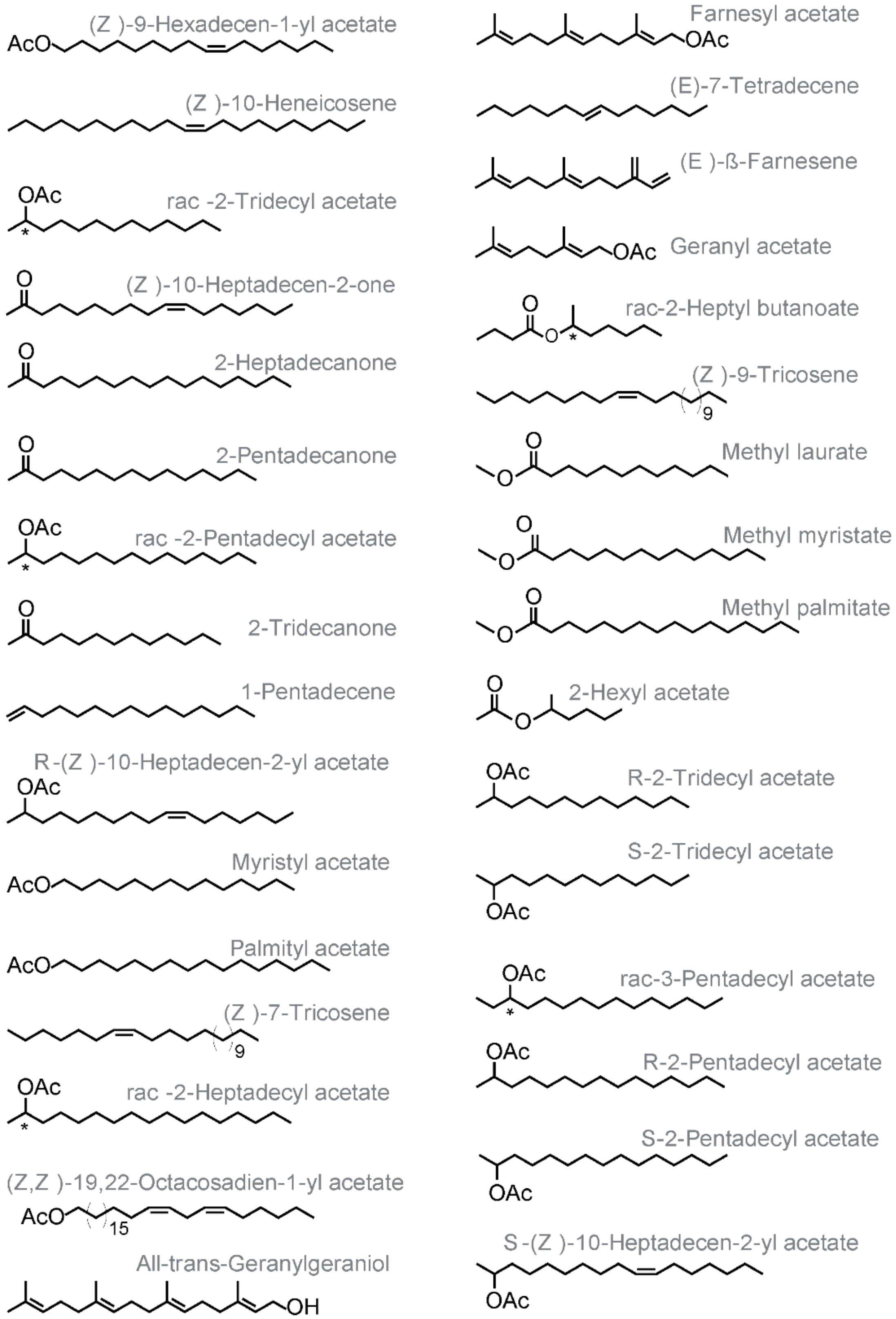

Figure S1. Chemical structure of the 32 tested compounds for which the receptors did not show significantly positive responses.
